# Consensus map integration and QTL meta-analysis narrowed a locus for yield traits to 0.7 cM and refined a region for late leaf spot resistance traits to 0.38 cM on linkage group A05 in peanut (*Arachis hypogaea* L.)

**DOI:** 10.1186/s12864-018-5288-3

**Published:** 2018-12-07

**Authors:** Qing Lu, Hao Liu, Yanbin Hong, Haifen Li, Haiyan Liu, Xingyu Li, Shijie Wen, Guiyuan Zhou, Shaoxiong Li, Xiaoping Chen, Xuanqiang Liang

**Affiliations:** Crops Research Institute, Guangdong Academy of Agricultural Sciences, South China Peanut Sub-Center of National Center of Oilseed Crops Improvement, Guangdong Provincial Key Laboratory of Crop Genetic Improvement, Guangzhou, 510640 China

**Keywords:** Peanut (*Arachis hypogaea* L.), Meta-analysis, Yield, Late leaf spot

## Abstract

**Background:**

Many large-effect quantitative trait loci (QTLs) for yield and disease resistance related traits have been identified in different mapping populations of peanut (*Arachis hypogaea* L.) under multiple environments. However, only a limited number of QTLs have been used in marker-assisted selection (MAS) because of unfavorable epistatic interactions between QTLs in different genetic backgrounds. Thus, it is essential to identify consensus QTLs across different environments and genetic backgrounds for use in MAS. Here, we used QTL meta-analysis to identify a set of consensus QTLs for yield and disease resistance related traits in peanut.

**Results:**

A new integrated consensus genetic map with 5874 loci was constructed. The map comprised 20 linkage groups (LGs) and was up to a total length of 2918.62 cM with average marker density of 2.01 loci per centimorgan (cM). A total of 292 initial QTLs were projected on the new consensus map, and 40 meta-QTLs (MQTLs) for yield and disease resistance related traits were detected on four LGs. The genetic intervals of these consensus MQTLs varied from 0.20 cM to 7.4 cM, which is narrower than the genetic intervals of the initial QTLs, meaning they may be suitable for use in MAS. Importantly, a region of the map that previously co-localized multiple major QTLs for pod traits was narrowed from 3.7 cM to 0.7 cM using an overlap region of four MQTLs for yield related traits on LG A05, which corresponds to a physical region of about 630.3 kb on the A05 pseudomolecule of peanut, including 38 annotated candidate genes (54 transcripts) related to catalytic activity and metabolic process. Additionally, one major MQTL for late leaf spot (LLS) was identified in a region of about 0.38 cM. BLAST searches identified 26 candidate genes (30 different transcripts) in this region, some of which were annotated as related to regulation of disease resistance in different plant species.

**Conclusions:**

Combined with the high-density marker consensus map, all the detected MQTLs could be useful in MAS. The biological functions of the 64 candidate genes should be validated to unravel the molecular mechanisms of yield and disease resistance in peanut.

**Electronic supplementary material:**

The online version of this article (10.1186/s12864-018-5288-3) contains supplementary material, which is available to authorized users.

## Background

Peanut (*Arachis hypogaea* L.) is a major oil and food crop that is cultivated widely in most tropical and subtropical areas of the world with global annual production of about 42 million tonnes (FAOSTAT, 2014). In breeding programs, a major objective is to increase yield, which is directly or indirectly influenced by pod, seed [1–3] and disease resistance related traits [4, 5]. Hence, the development of high-yield and disease resistant varieties is still the best approach to increase peanut production. However, these traits are typical complex quantitative traits, which make genetic improvement using traditional breeding methods very challenging.

Quantitative trait locus (QTL) analysis provides location information for target traits and can be applied in molecular breeding using marker-assisted selection (MAS). During the past 10 years, the numbers of reported yield and disease resistance related QTLs have increased tremendously in peanut (https://www.peanutbase.org/). However, only a few marker-trait associations have been used for MAS [6] because the effect and consistency of QTLs across different genetic backgrounds and environments are key factors in the successful use of the QTLs in MAS breeding. In general, most of the reported QTLs for yield and disease resistance were mapped using a single genetic background under a limited number of environments [2, 3 4 5]. Many of these QTLs may reduce or even not provide a consistent phenotypic effect when introduced to a new genetic background under a different environment because of unfavorable epistatic interactions [7]. Thus, it is important to predict the usefulness of consensus QTLs for MAS in the individual genetic background of the target species in a particular study.

QTL meta-analysis has been used to identify consensus QTLs across multiple studies for their consistency of location and effect across different genetic backgrounds and environments, as well as to refine QTL positions on a consensus map [8]. QTL meta-analysis requires two necessary conditions, namely, a consensus map with high-density markers [9, 10] and independent QTLs for the same trait identified from different genetic backgrounds and environments [8]. Consensus QTLs obtained from meta-analysis based on a lot of QTLs related to a target trait at a 95% confidence interval (CI) are called meta-QTLs (MQTLs). MQTLs with the smallest CIs and consistent and large effects on a trait have been used efficiently in MAS crop breeding because of multiple QTL integrations, such MQTLs have been applied for yield related traits in rice [11, 12], disease resistance in maize [13, 14], flowering time in winter wheat [15], and seed quality in soybean [16]. However, to date, only a few MQTLs for peanut yield and disease resistance have been reported [17], limiting the wide application of MAS to these traits.

In this study, we constructed a high-density consensus map of peanut and used it to carry out a QTL meta-analysis to identify MQTLs for yield and disease resistance traits using BioMercator V4.2 [18]. The purpose of the study was (1) to construct a new consensus genetic map with high-density markers; (2) to identify lots of MQTLs with large effects and small CIs relative to the initial QTLs; (3) to refine the initial QTLs for candidate gene prediction; and (4) to provide markers of the MQTLs for possible use in MAS.

## Methods

### Literature review and QTL collection

Peanut pod and seed related traits, such as 100 pod weight (100 PW), 100 seed weight (100SW), shelling percentage (SP), pod length (PL), pod width (PW), seed length (SL), and seed width (SW) and disease resistance traits, for example tomato spotted wilt virus (TSWV), early leaf spot (ELS), and late leaf spot (LLS) are greatly limit the increase of peanut yield per unit. In this study, we used a meta-analysis to integrate consensus QTLs for MAS breeding. The QTL information was collected from seven reports published in the recent years involving QTL mapping for yield and disease resistance traits. We used 292 of 305 initial QTLs to identify MQTLs and refine QTL positions (Additional file 1: Table S1).

### Consensus map integration

Based on a previous integrated consensus map of cultivated peanut [10], we constructed a new consensus map with high-density markers using BioMercator V4.2 with default parameters [18]. All the markers from eight studies (Additional file 1: Table S1) were used to develop the consensus genetic map. Linkage groups (LGs) connected with fewer than two common markers were excluded before construction of the consensus map. Polymorphic loci of each marker were visualized on the A- and B-subgenomes using RCircos in R (https://www.r-project.org/) [19].

### QTL projection on the consensus map

Projection of the initial QTLs on the high-density consensus map was based on LOD scores, phenotypic variation explained by each QTL, CIs, and QTL flanking marker positions. For the QTLs that lacked flanking markers and CIs, the 95% CIs of the initial QTLs on the original maps were estimated as: CI = 530 / (*N* × *R*^2^) (1) or CI = 163 / (*N* × *R*^2^) (2) [20], where *N* is the population size and *R*^2^ is the proportion of the phenotypic variation explained by the QTL. Eq. (1) was applied to QTL studies that used backcross and F_2_ mapping populations, and eq. (2) was used when the QTLs were identified using recombinant inbred line mapping populations. The position of the projected QTL was determined using a simple scaling rule between the original QTL flanking marker interval and the corresponding interval on the consensus map. For the projected QTL, the new CI on the consensus LGs was approximated with a Gaussian distribution around the most likely QTL position. All the QTL projections were performed using BioMercator V4.2 [18].

### QTL meta-analysis

Based on the integrated consensus map and initial QTL projections, we performed a QTL meta-analysis according to the QTL clusters on each LG of the consensus map to identify MQTLs using BioMercator V4.2 [18] and algorithms from the MetaQTL software [21]. Two steps are required for a successful meta-analysis. In step 1, the projected QTLs are clustered on each LG with default parameters. Then, five criteria, Akaike information criterion (AIC), AIC correction, AIC 3 candidate model, Bayesian information criterion, and average weight of evidence, are used to determine the number of potential MQTLs for the next step. In step 2, the MQTLs are generated in accordance with the best model of step 1 [21]. In our meta-analysis, the QTL model with the lowest AIC value was used to determine the number of MQTLs on each LG [22, 23]. Finally, the position and the 95% CI of each MQTL were calculated, and the flanking markers of the MQTL were selected for application in MAS breeding.

### Detecting candidate genes

The flanking markers of the CIs of the identified MQTLs were used to search for candidate genes. The genome assembly of cultivated peanut was used as the reference genome (https://www.peanutbase.org/). Then, the sequences of the flanking markers were mapped to the reference genome, and the physical positions were obtained. Finally, the candidate genes were identified using GBrowse on the PeanutBase website (https://www.peanutbase.org/).

## Results and discussion

### Overview of QTLs and consensus map integration

**Fig. 1:**
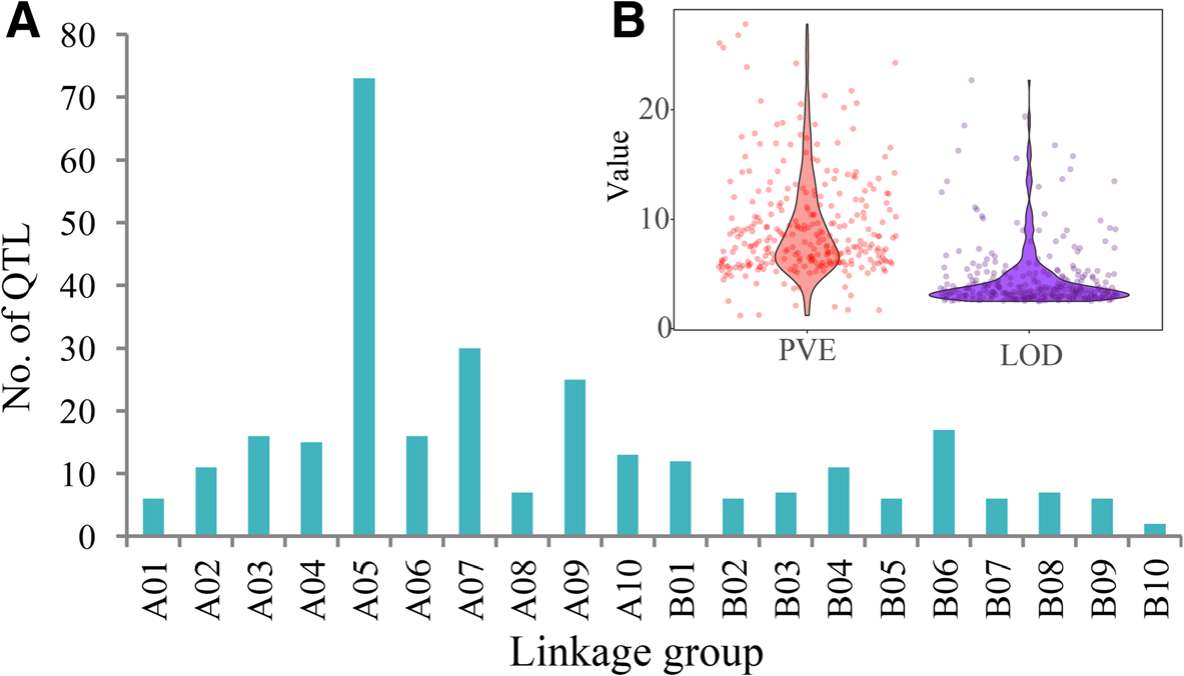
Summary of initial QTLs. **a** Distribution of all the initial QTLs on 20 LGs. **b** Phenotypic variance and LOD value of each initial QTL. PVE, phenotypic variance explained by the QTL

To collect genetic map and QTL information for peanut yield and disease resistance traits, we mined PeanutBase (https://www.peanutbase.org/) and recent reports in the literature up to 2017 (Additional file 1: Table S1). Eight individual genetic maps and one consensus map, which together contained a total of 8581 markers, were used to construct a new consensus genetic map. We identified 292 of 305 initial QTLs related to yield and disease resistance for use in the QTL meta-analysis (Additional file 1: Table S1). The initial QTLs were distributed on all 20 LGs; the highest number was on A05, followed by A07 and A09 (Fig. 1a; Additional file 2: Figure S1). The phenotypic variance explained by the initial QTLs ranged from 1.2 to 27.8% and the LOD value varied from 2.5 to 22.7 (Fig. 1b).

**Fig. 2:**
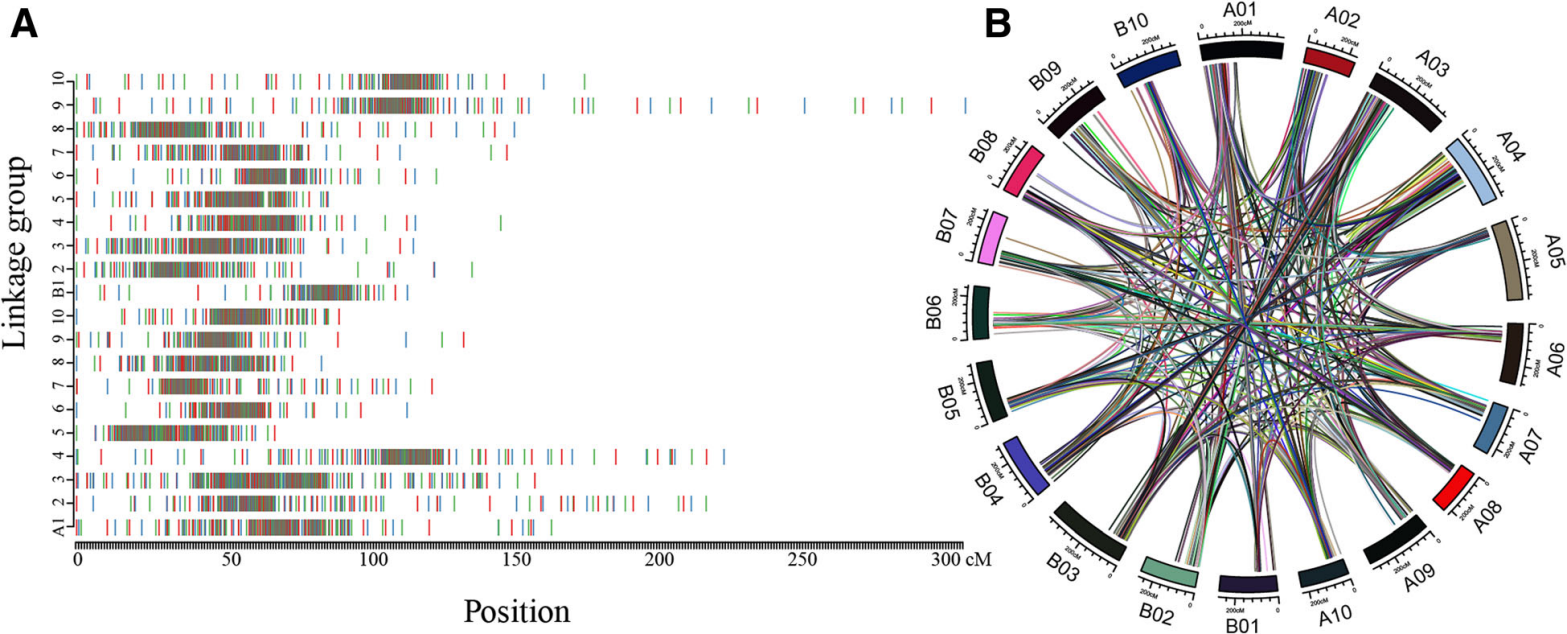
Overview of the new integrated consensus map. **a** Distribution of 5874 loci on 20 linkage groups (LGs); **b** Distribution of markers with more than two loci among the 20 LGs. Homologous marker loci are connected by lines

A new integrated consensus map was constructed using BioMercator V4.2 [18] based on nine previously published genetic maps (Additional file 1: Table S1). The integrated consensus map was up to 2918.62 centimorgan (cM) long with an average marker density of 2.01 loci per cM and 20 LGs on which 5874 loci were mapped (Table 1; Fig. 2a; Additional file 3: Table S2). In 2010, a composite linkage map with 175 SSR markers was published; it was 885.4 cM long with an average marker density of 0.19 loci per cM [9]. In 2013, a consensus map covering 2651 cM with 3693 loci that were mapped to 20 LGs was integrated with the 2010 and other maps, taking the marker density up to 1.39 loci per cM [10]. In the present study, the new consensus map contains more markers and higher marker density than the previous consensus genetic map, making it more suitable for our QTL meta-analysis.

**Table 1:** Details of the new integrated consensus map constructed in this study

Linkage group	No. of locus	cM	Density (locus/cM)
A01	393	165.94	2.37
A02	237	219.98	1.08
A03	379	160.11	2.37
A04	337	226.26	1.49
A05	380	69.23	5.49
A06	289	115.49	2.50
A07	239	124.19	1.92
A08	223	85.60	2.61
A09	314	135.29	2.32
A10	231	91.75	2.52
B01	277	115.65	2.40
B02	268	138.21	1.94
B03	380	117.70	3.23
B04	292	148.19	1.97
B05	287	87.83	3.27
B06	250	125.71	1.99
B07	250	150.36	1.66
B08	247	153.02	1.61
B09	300	310.64	0.97
B10	301	177.47	1.70
Total	5874	2918.62	2.01

Comparison of all marker loci indicated that 889 markers mapped to more than two loci among different LGs of the corresponding A- and B-subgenomes or within A- and B-subgenomes (Fig. 2b; Additional file 4: Figure S2). Cultivated peanut is an allotetraploid (AABB-type genome; 2n = 4× = 40), probably derived from its diploid ancestors (*Arachis duranensis*, AA-type genome and *Arachis ipaensis*, BB-type genome). The two ancestor genome sequences showed collinearity of contigs and high sequence identity (≥99%), and analysis of their chromosomal structure and synteny indicated that most pseudomolecules had a one-to-one correspondence of collinearity or inversion between the two species [24]. This result may be a reasonable explanation for the distribution of different marker loci on different LGs in the new integrated consensus map.

### Identification of MQTLs for yield and disease resistance related traits

**Fig. 3:**
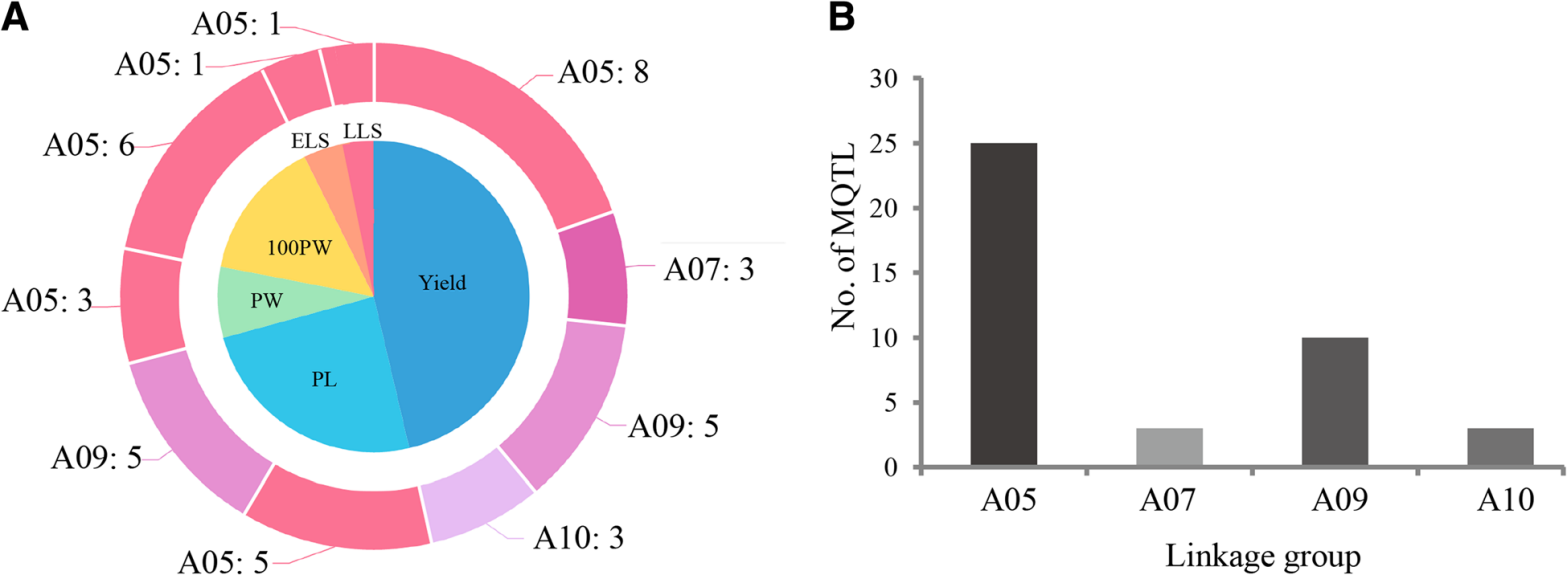
MQTLs for yield and disease resistance related traits. **a** MQTLs for different traits distributing on different linkage groups (LGs). **b** Number of MQTLs on four LGs. PL, pod length; PW, pod width; 100PW, 100 pod weight; ELS, early leaf spot; LLS, late leaf spot

**Fig. 4:**
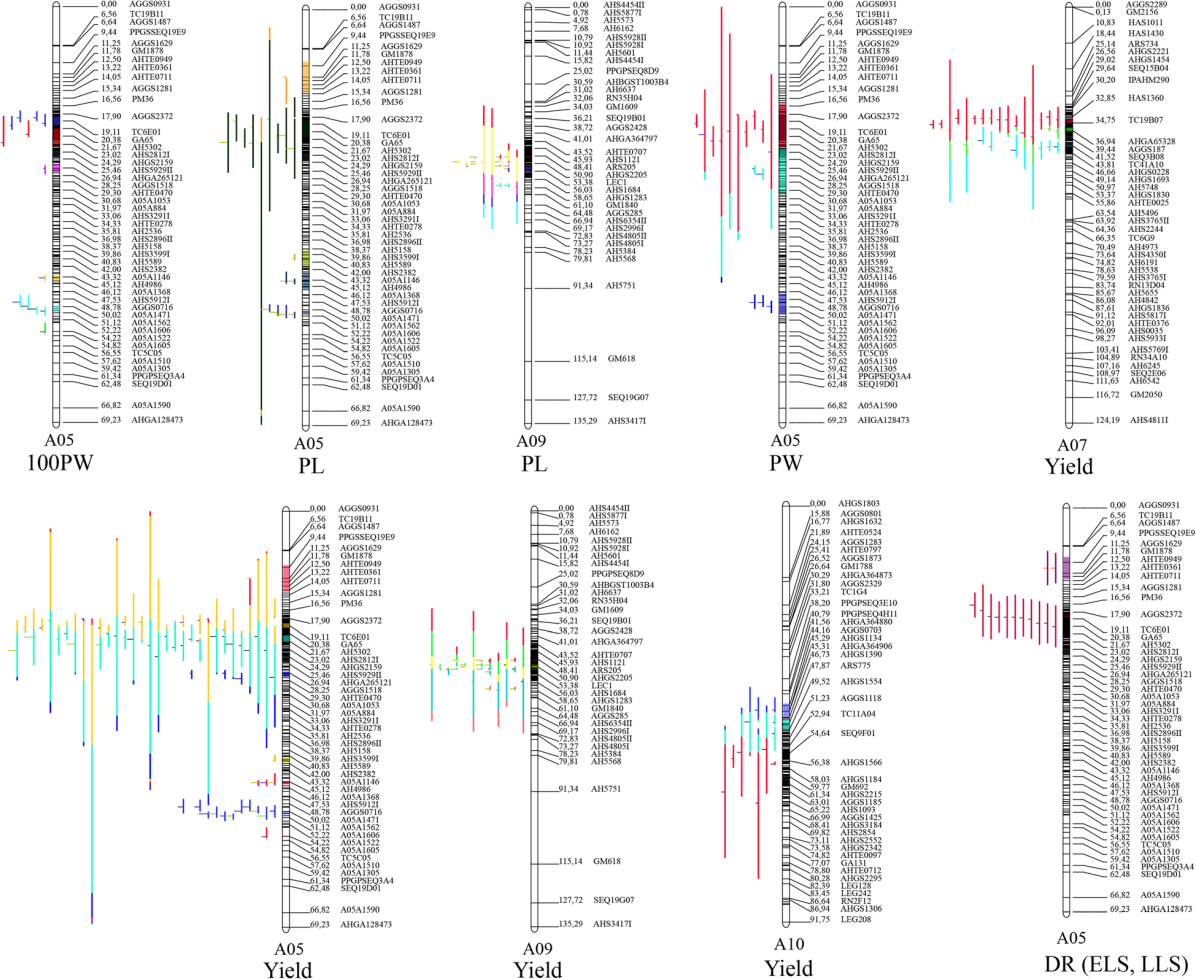
MQTL hotspots for yield and disease resistance related traits. PL, pod length; PW, pod width; 100PW, 100 pod weight; DR, disease resistance; ELS, early leaf spot; LLS, late leaf spot

**Fig. 5:**
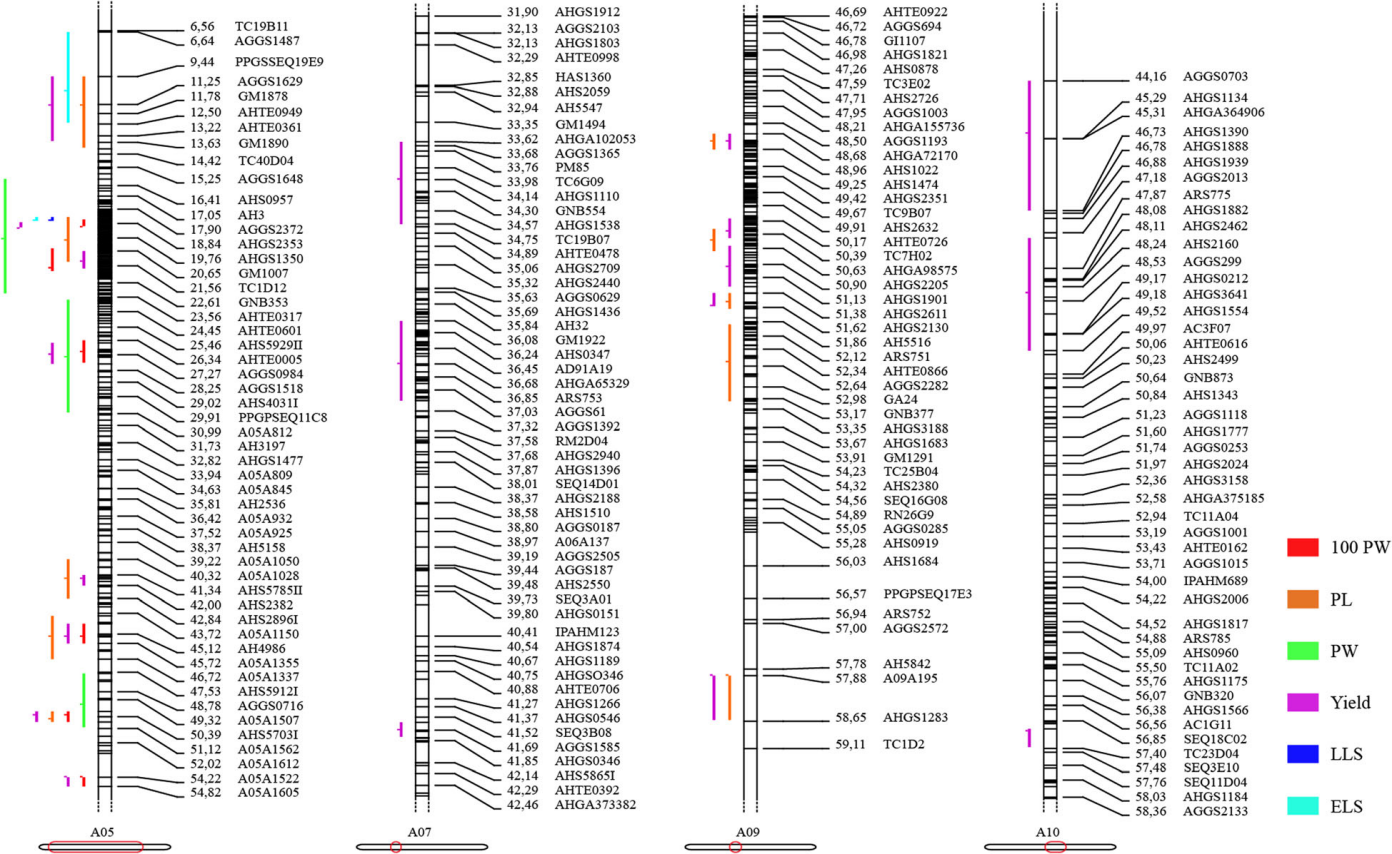
MQTL hotspots in each linkage group of the new integrated consensus map. PL, pod length; PW, pod width; 100PW, 100 pod weight; ELS, early leaf spot; LLS, late leaf spot

The precise location of QTLs and their application in MAS are affected by their genetic backgrounds and environments. QTL meta-analysis helps to identify the most precise and concise QTLs based on previous different studies for single traits or comprehensive agronomic traits, such as yield and disease resistance, that can be further pursued for MAS or to predict candidate genes. In this study, yield related traits were defined as 100PW, 100SW, SP, PL, PW, SL, and SW, and disease resistance traits were separated as resistance to TSWV, ELS, and LLS. Our literature survey identified a set of 292 initial QTLs for yield and disease resistance related traits that were projected onto the new consensus map. Then, the AIC values, which we consider to be the best QTL model to determine the number of MQTLs, and the 95% CIs, were calculated. A total of 40 independent MQTLs were identified on LGs A05, A07, A09, and A10. The genetic intervals of 20 of the MQTLs were less than 1 cM, and most of the intervals were narrower than their respective initial QTL (Additional file 5: Table S3). Among them, MQTLs for yield were most abundant, followed by MQTLs for PL (Fig. 3a). The number of MQTLs distributed on each LG varied from 3 to 24 (Fig. 3b), and most of the identified MQTLs had hotspots on each LG (Fig. 4), for example, A05 had the highest number of MQTL hotspots for the yield and disease resistance related traits (Fig. 5). This may be because the highest number of initial QTLs was identified on A05 (Additional file 2: Figure S1), or because A05 has higher marker density than the other LGs (Table 1), making the detection of MQTLs easier.

The MQTLs on A05 formed eight clusters (CMQTLs) with two or more MQTL overlapping regions (Additional file 6: Table S4). The sequences of the markers flanking the CMQTLs were used in BLAST searches against the reference genome assembly. The physical positions that were obtained varied from 581.5 kb to 6115.7 kb (Additional file 6: Table S4). These flanking markers of the narrowed genetic intervals of the MQTLs and the small physical intervals of the CMQTLs may be useful in MAS (Additional file 5: Table S3; Additional file 6: Table S4).

### MQTL hotspots for yield related traits and identification of candidate genes

**Fig. 6:**
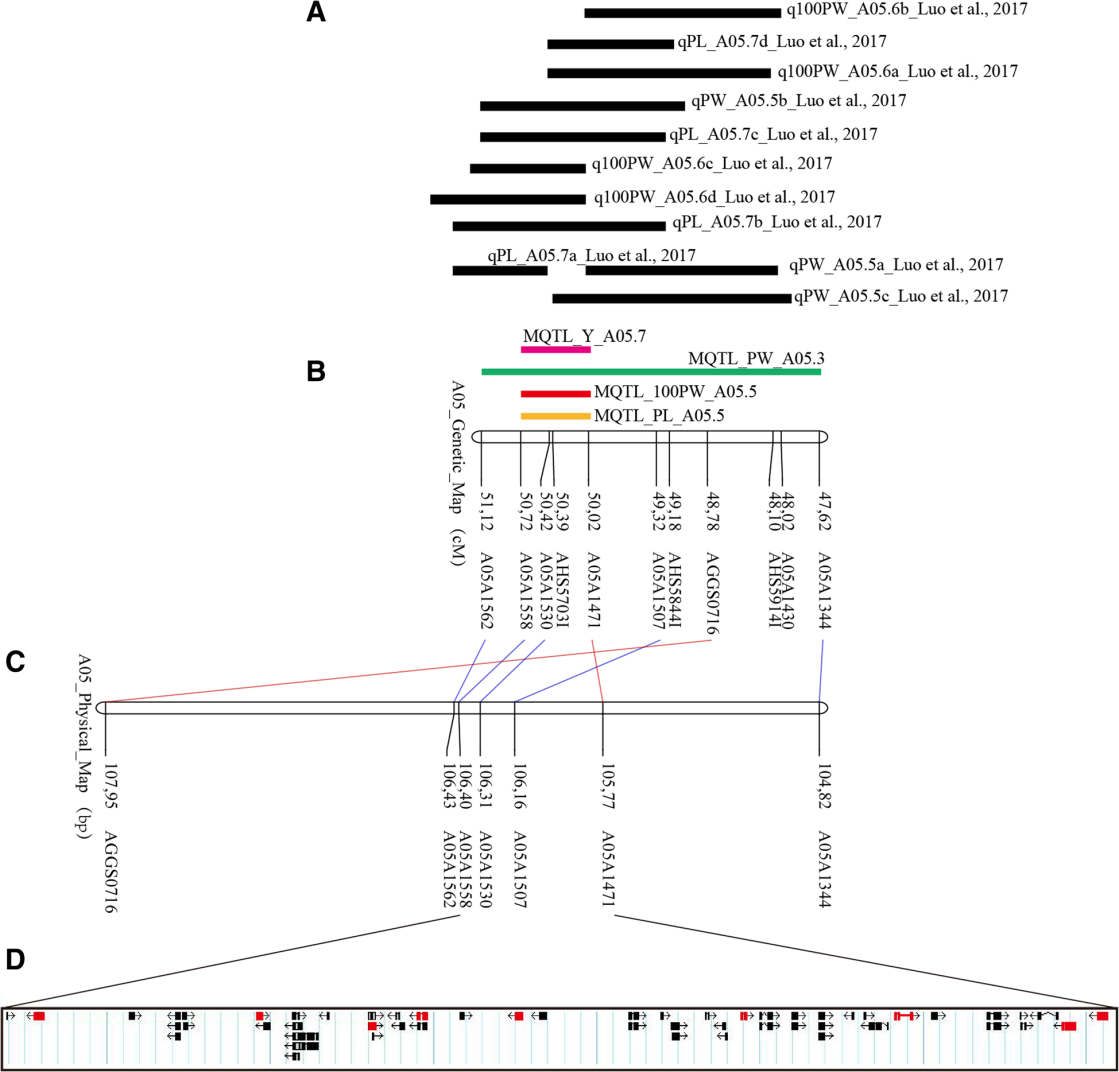
Fine mapping of MQTLs for yield related traits and identification of candidate genes. **a** Co-localization of initial major QTLs for pod size and weight in multiple environments [25]. **b** MQTL for yield related traits on linkage group A05. **c** Physical map of the narrowed MQTL interval on pseudomolecule A05 of *Arachis hypogaea*. **d** Identification of candidate genes in the narrowed MQTL interval. Red boxes represent homologous genes that were identified in pseudomolecule A05 of *Arachis duranensis* V14167 in a previous report [25]

A previous report has indicated that multiple major QTLs for pod size and pod weight were co-localized to a 3.7 cM interval on LG A05 [25] (Fig. 6a), which harbored three major QTLs for pod size and weight traits, namely, *qPLA05.7* for PL, *qPWA05.5* for PW, and *qHPWA05.6* for 100PW. Moreover, these three QTLs explained a significant proportion of the phenotypic variance; 16.89–27.84% for PL, 13.73–14.12% for PW, and 13.75–26.82% for 100PW [25]. Based on the QTL meta-analysis, this region was narrowed to a 0.7 cM interval that contained four overlapping MQTLs, namely, MQTL_Y_A05.7, MOTL_PW_PA05.3, MQTL_100PW_A05.5, and MQTL_PL_A05.5, (Fig. 6b), and explained 19–31% of the phenotypic variance (Additional file 5: Table S3).

Moreover, the 0.7 cM interval corresponded to a 630.3 kb physical region of the A05 pseudomolecule (Fig. 6c), which contains 38 candidate genes (54 different transcripts), among which eight encode unknown proteins, while others have homologs with a variety of protein functions (Fig. 6d; Additional file 7: Table S5). Importantly, nine of these proteins are homologous to proteins previously identified in the A05 pseudomolecule of *A. duranensis* V14167 [25] (Fig. 6d; Additional file 7: Table S5). In addition, 37 of the 54 transcripts were assigned at least one gene ontology (GO) term, including binding and catalytic activity under the molecular function category, and metabolic and cellular process under the biological process category (Additional file 8: Figure S3a). These results highlight the importance of this interval as a target for improving yield related traits through MAS, as well as providing an opportunity for QTL fine mapping and the validation of candidate genes.

### MQTL hotspots for disease resistance related traits and identification of candidate genes

**Fig. 7:**
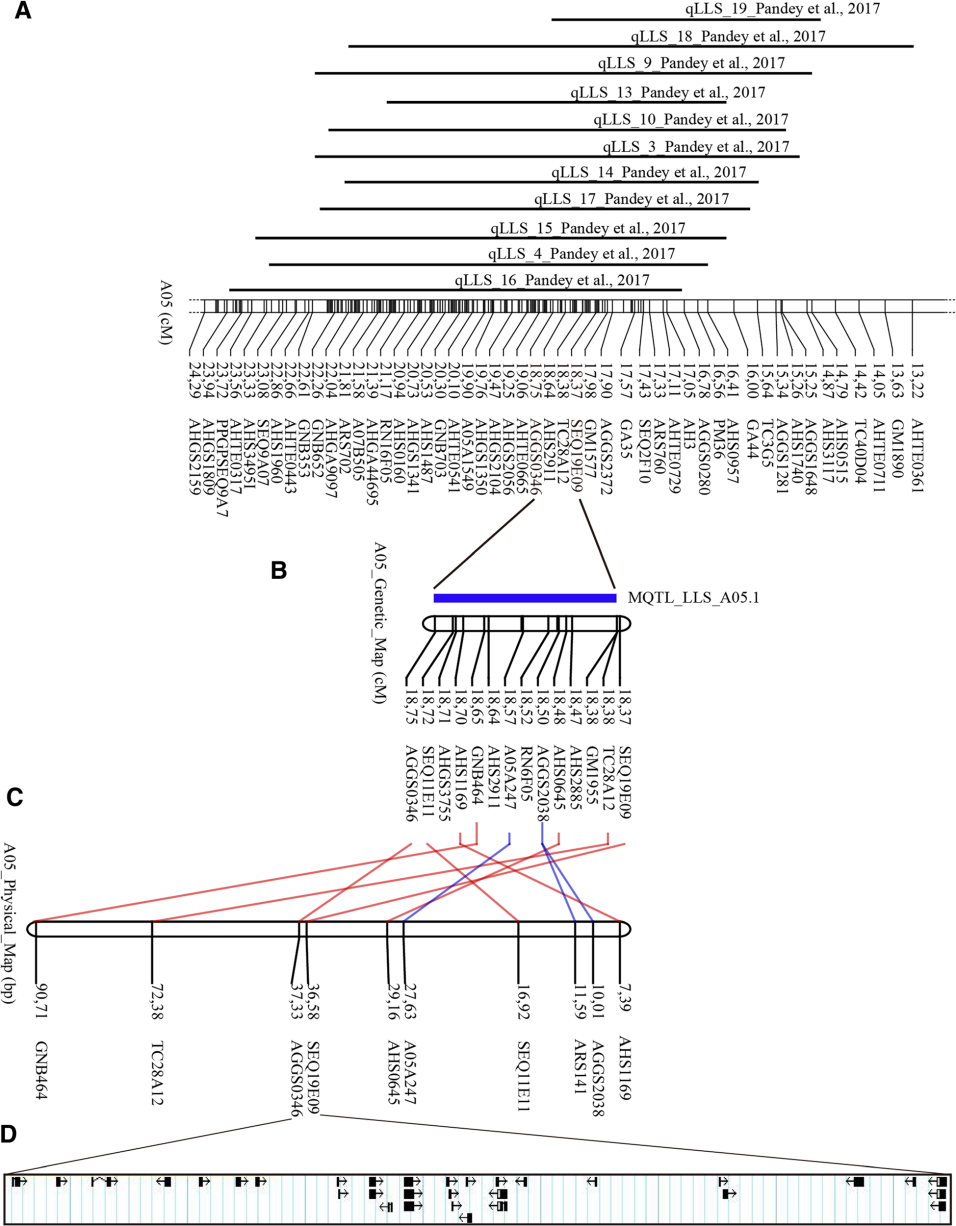
Fine mapping of MQTLs for late leaf spot and identification of candidate genes. **a** Co-localization of initial QTLs for late leaf spot across different environments [4]. **b** MQTL for late leaf spot on linkage group A05. **c** Physical map of the narrowed MQTL interval on pseudomolecule A05 of *Arachis hypogaea*. **d** Identification of candidate genes in the physical map interval

TSWV, ELS, and LLS are three of the most serious diseases in peanut worldwide. They not only cause huge annual yield losses but also affect seed and oil quality [4]. High production costs and environment pollution are challenges to peanut growers because of the over-reliance on agricultural chemicals that are used in traditional farming to control these diseases. In recent years, molecular breeding techniques, such as MAS, have proven to be effective in genetic improvement of peanuts for disease resistance [26]. Thus, identification of consensus QTLs for disease resistance is particularly important. In this study, we used a total of 42 initial QTLs identified by Pandey et al. [4] to identify MQTLs. Finally, we detected two MQTLs for disease resistance related traits on LG A05 using meta-analysis (Additional file 5: Table S3). One is MQTL_ELS_A05.1 for ELS and the other is MQTL_LLS_A05.1 for LLS (Fig. 3a, Figs. 4, and 5; Additional file 5: Table S3). Previously, 11 QTLs for LLS resistance were mapped and overlapped on LG A05 across multiple environments [4] (Fig. 7a), and the MQTL (MQTL_LLS_A05.1) for LLS was detected between markers SEQ19E09 and AGGS0346, which flanked a narrow 0.38 cM interval (Fig. 7b). The flanking markers of the MQTL for LLS could be useful for MAS breeding to improve the resistance to LLS in peanut.

The BLAST searches indicated that the corresponding physical interval was about 742.3 kb on the A05 pseudomolecule (Fig. 7c; Additional file 6: Table S4), which contained 26 candidate genes (30 different transcripts) (Fig. 7d; Additional file 9: Table S6). Some of these genes encode homologs of known proteins related to stress or disease resistances. For example, *Arahy.AXI81X* encodes a F-box/RNI-like superfamily protein that plays a role in the control of disease resistance in rice [27], and *Arahy.CBXD69* and *Arahy.MF7AUF* encode a protein phosphatase 2C (PP2C)-like domain, which plays an important role in the regulation of disease resistance through activation of the defense response in plants [28, 29]. Moreover, *Arahy.M3KMZQ*, *Arahy.SQ144R* and *Arahy.FCT2UL* annotated a zinc knuckle family protein, which is involved in plant disease resistance [30]. In addition, *Arahy.37S24P* annotated as a AP2/EREBP (APETALA2/ ethylene-responsive element binding protein) transcription factor, which can enhance disease resistance and salt tolerance [31, 32].The associated GO terms suggested that most of these genes have catalytic and binding activity and are involved in metabolic and cellular biological process (Additional file 8: Figure S3). Our results provide a set of candidate genes that potentially play crucial roles in peanut disease resistance. Validation of the biological functions of these genes will be of interest in future studies.

## Conclusions

Meta-analysis of QTLs for yield and disease resistance related traits is an effective approach to integrate consensus QTLs and refine initial QTLs. In this study, we identified a set of 40 MQTLs with narrowed genetic intervals that could be helpful in MAS. Some of these MQTLs are clustered at different hotspots on LG A05. Combined with a physical map (https://www.peanutbase.org/), the flanking markers defining the CMQTLs were used to search a limited list of candidate genes related to yield and disease resistance traits. These genes are valuable targets for biological validation in the future.

## Additional files

Additional file 1: **Table S1.** Details of previous mapping populations used for consensus map construction and QTL meta-analysis. (XLSX 14 kb)

Additional file 2: **Figure S1.** Distribution of all the initial QTLs on the linkage groups of the integrated consensus map. (DOCX 671 kb)

Additional file 3: **Table S2.** Description of the new integrated consensus map. (XLSX 549 kb)

Additional file 4: **Figure S2.** Distribution of different loci on each linkage group of the integrated consensus map. (DOCX 422 kb)

Additional file 5: **Table S3.** MQTLs for yield and disease resistance identified by meta-analysis. (XLSX 17 kb)

Additional file 6: **Table S4.** Summary of MQTL hotspots on linkage group A05. (XLSX 14 kb)

Additional file 7: **Table S5.** Summary of 38 candidate genes of cluster CMQTL7 for yield related traits. (XLSX 16 kb)

Additional file 8: **Figure S3.** Enrichment analysis of gene ontology terms for the candidate genes for yield and late leaf spot. (DOCX 174 kb)

Additional file 9: **Table S6.** Summary of 26 candidate genes of cluster CMQTL9 for late leaf spot. (XLSX 13 kb)

## Abbreviations

100PW: 100 pod weight
100SW: 100 seed weight
cM: centimorgan
CMQTL: Cluster of MQTL
ELS: Early leaf spot
LG: Linkage group
LLS: Late leaf spot
MAS: Marker-assisted selection
MQTL: Meta-QTL
PL: Pod length
PW: Pod width
QTL: Quantitative trait loci
SL: Seed length
SP: Shelling percentage
SW: Seed width
TSWV: Tomato spotted wilt virus
