# Supplementary material for: Consensus map integration and QTL meta-analysis narrowed a locus for yield traits to 0.7 cM and refined a region for late leaf spot resistance traits to 0.38 cM on linkage group A05 in peanut (Arachis hypogaea L.)
Source: BMC Genomics. 2018 Dec 7;19:887. doi: 10.1186/s12864-018-5288-3 (PMC6286586; doi:10.1186/s12864-018-5288-3)
Supplement: Supplementary file 2 — Figure S1. Distribution of all the initial QTLs on the linkage groups of the integrated consensus map. (DOCX 671 kb) [file 12864_2018_5288_MOESM2_ESM.docx]

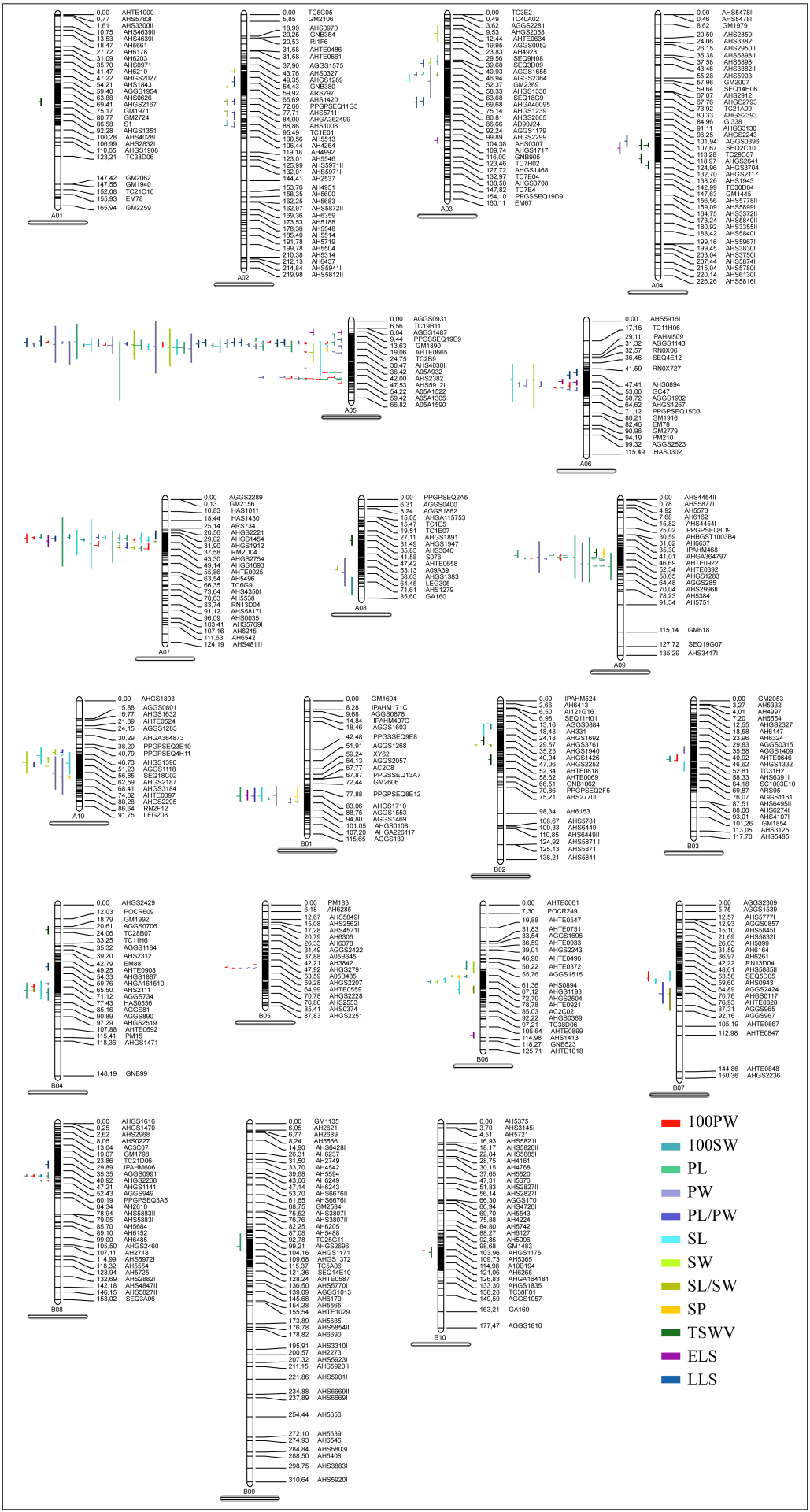


**Figure S1. Distribution of all the initial QTLs on the linkage groups of the integrated consensus map.** 100PW: 100 pod weight; 100SW: 100 seed weight; PL: pod length; PW: pod width; SL: seed length; SW: seed width; SP: shelling percentage; SP: shelling percentage; ELS: early leaf spot; LLS: late leaf spot.
