# Supplementary material for: Consensus map integration and QTL meta-analysis narrowed a locus for yield traits to 0.7 cM and refined a region for late leaf spot resistance traits to 0.38 cM on linkage group A05 in peanut (Arachis hypogaea L.)
Source: BMC Genomics. 2018 Dec 7;19:887. doi: 10.1186/s12864-018-5288-3 (PMC6286586; doi:10.1186/s12864-018-5288-3)
Supplement: Supplementary file 4 — Figure S2. Distribution of different loci on each linkage group of the integrated consensus map. (DOCX 422 kb) [file 12864_2018_5288_MOESM4_ESM.docx]

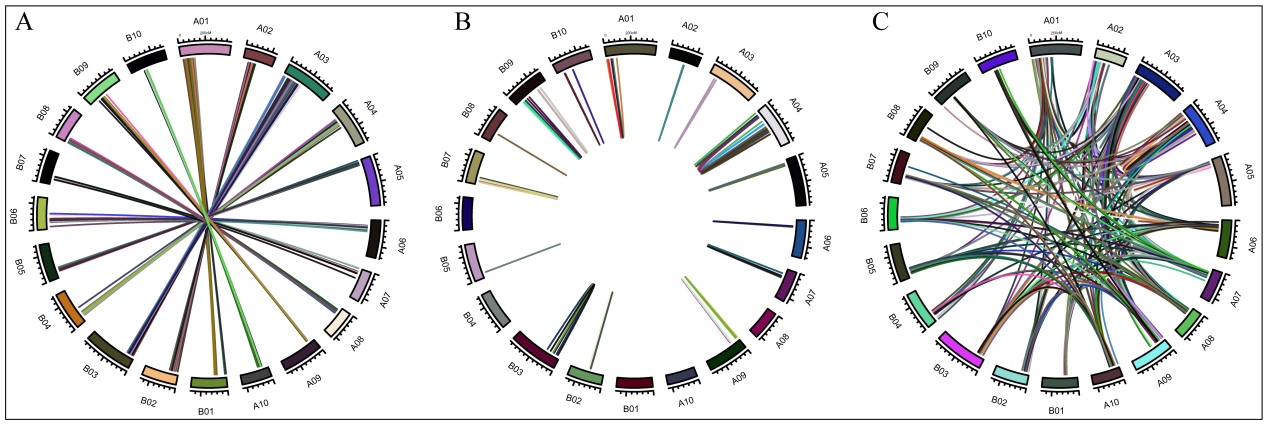


**Figure S2. Distribution of different loci on each linkage group of the integrated consensus map.** (A) The loci showed a one-to-one correspondence between A- and B-subgenomes; (B) The loci located on the same A- and B-subgenomes; (C) The loci located on different A- and B-subgemomes.
