# Supplementary material for: Consensus map integration and QTL meta-analysis narrowed a locus for yield traits to 0.7 cM and refined a region for late leaf spot resistance traits to 0.38 cM on linkage group A05 in peanut (Arachis hypogaea L.)
Source: BMC Genomics. 2018 Dec 7;19:887. doi: 10.1186/s12864-018-5288-3 (PMC6286586; doi:10.1186/s12864-018-5288-3)
Supplement: Supplementary file 8 — Figure S3. Enrichment analysis of gene ontology terms for the candidate genes for yield and late leaf spot. (DOCX 174 kb) [file 12864_2018_5288_MOESM8_ESM.docx]

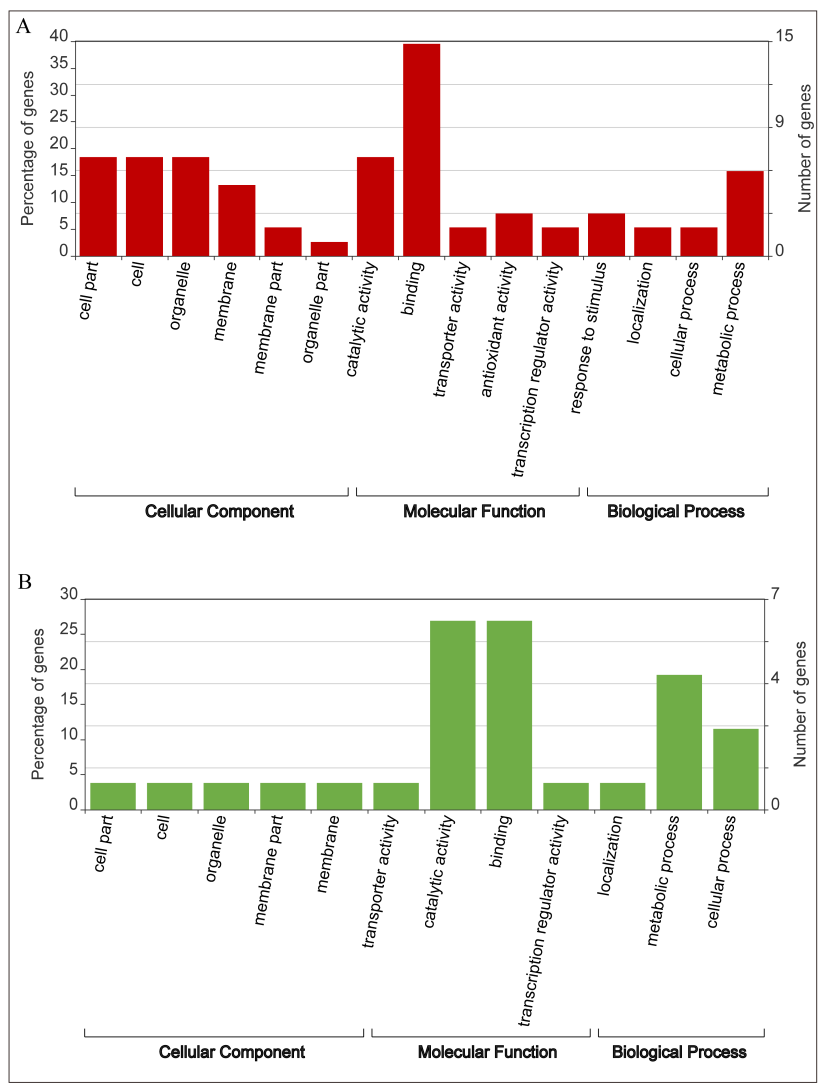


**Figure S3. Enrichment analysis of gene ontology terms for the candidate genes for yield and late leaf spot.** (A) GO term of the candidate genes related to yield traits; (B) GO term of the candidate genes for late leaf spot.
